# Impurites of Salicylic Acid

**Published:** 1890-03-22

**Authors:** 


### IMPURITIES OF SALICYLIC ACID.

The *British Medical Journal* calls attention to an important point in practical therapeutics. Salicylic acid and its compounds are now very extensively used in the treatment of rheumatism and other diseases. Certain experiments of Professor Charteris, therefore, on impurities of that drug, cannot fail to be of interest to the general practitioner. Briefly stated, the British Pharmacopœia allows salicylic acid to be prepared in two ways: (1) artificially from carbolic acid; (2) from natural salicylates, such as oil of winter green, or of sweet birch. Of the artificial preparations, ten grains of the acid, and eighteen of the sodium salt, killed a rabbit. Of the natural, thirty grains of salicin, ten of the acid, and thirty-two of the salt, did not injure a rabbit of two and a-half pounds. The poisonous impurity in the artificial preparations has been separated, and one grain killed a rabbit of two pounds. The injurious effects of the salicylates are chiefly headache, singing in the ears, and delirium. These investigations are likely to throw discredit on the present artificial preparations, and to open up a market for an absolutely pure drug.

